# Supplementary material for: Primary cilia regulate hematopoietic stem and progenitor cell specification through Notch signaling in zebrafish
Source: Nat Commun. 2019 Apr 23;10:1839. doi: 10.1038/s41467-019-09403-7 (PMC6478842; doi:10.1038/s41467-019-09403-7)
Supplement: Supplementary file 1 — Supplementary Information [file 41467_2019_9403_MOESM1_ESM.pdf]

Primary cilia regulate hematopoietic stem and progenitor cell specification through Notch signaling in zebrafish

Liu et al.

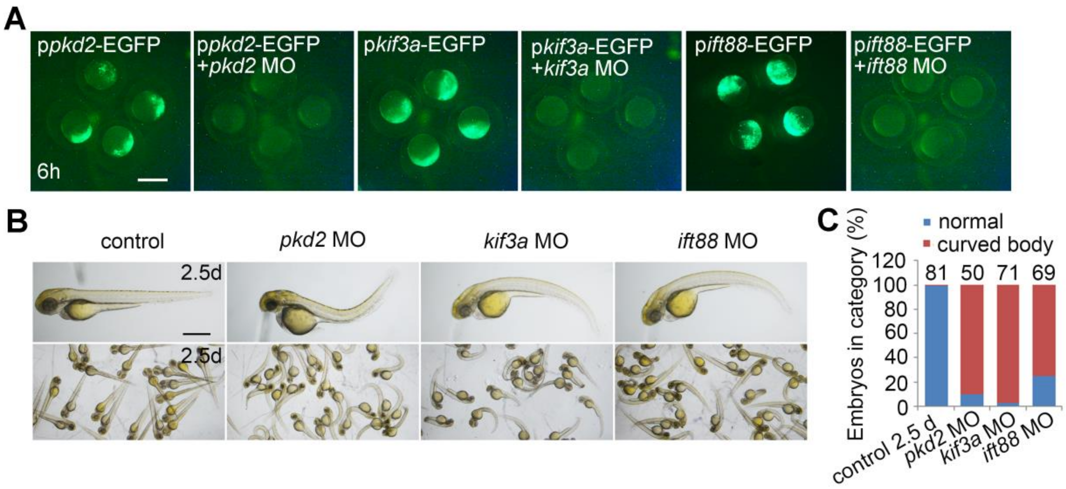

Supplementary Figure 1. Validation of the efficiency of cilia-related MOs. (A) The validation of the efficiency of *pkd2*, *kif3a* and *ift88* MOs through co-injection of pEGFP-N1-*pkd2*/*kif3a*/*ift88* (p*pkd2*/*kif3a*/*ift88*-EGFP) and *pkd2*/*kif3a*/*ift88* MOs at one-cell stage embryos. (B, C) The phenotypes of control embryos, *pkd2*, *kif3a* and *ift88* morphants at 2.5 dpf with quantification. Scale bars, 500 μm.

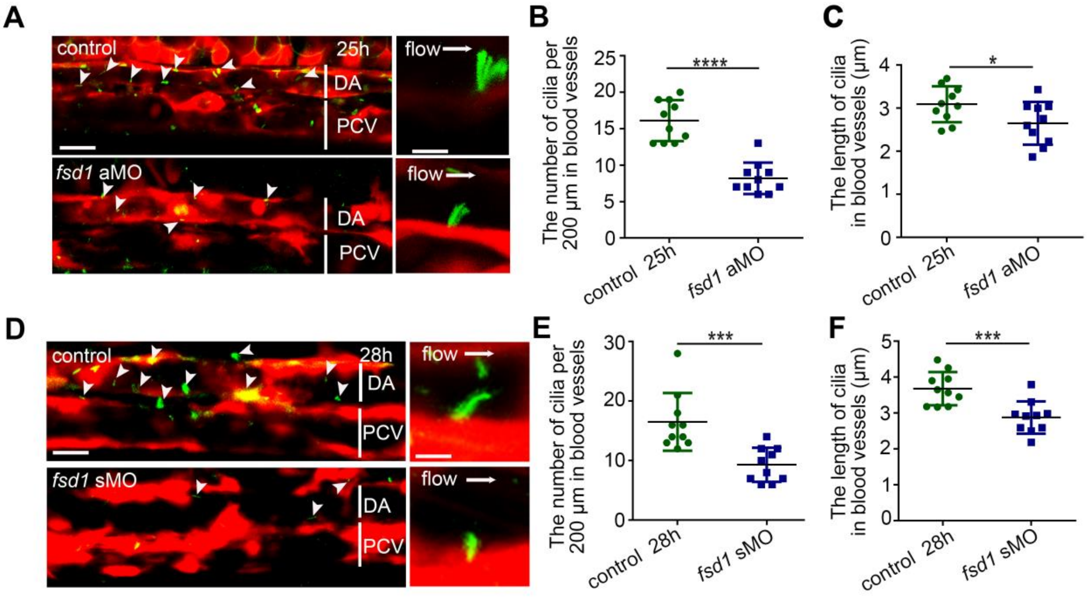

Supplementary Figure 2. Primary cilia are impaired in *fsd1* morphants. (A) Confocal imaging of endothelial primary cilia (white arrowheads) in blood vessels in the AGM region in control and *fsd1* aMO-injected embryos within Tg(βact::Arl13b–GFP/*kdrl*:mCherry) transgenic line at 25 hpf. Scale bars, 20 µm (left panel) and 5 µm (right panel). (B, C) Quantification of primary cilia number and length in control and *fsd1* aMO-injected embryos. (D-F) Confocal imaging of endothelial primary cilia (white arrowheads) in control and *fsd1* sMO-injected embryos with quantification of cilia number (E) and length (F). White bars denote DA or PCV region. Scale bars, 20 µm (left panel) and 5 µm (right panel). White arrows mark blood flow direction. Error bars, mean ± s.d., n=10 embryos. ns, non-significant, *P < 0.05, ***P < 0.001, ****P < 0.0001, Student's t-test.

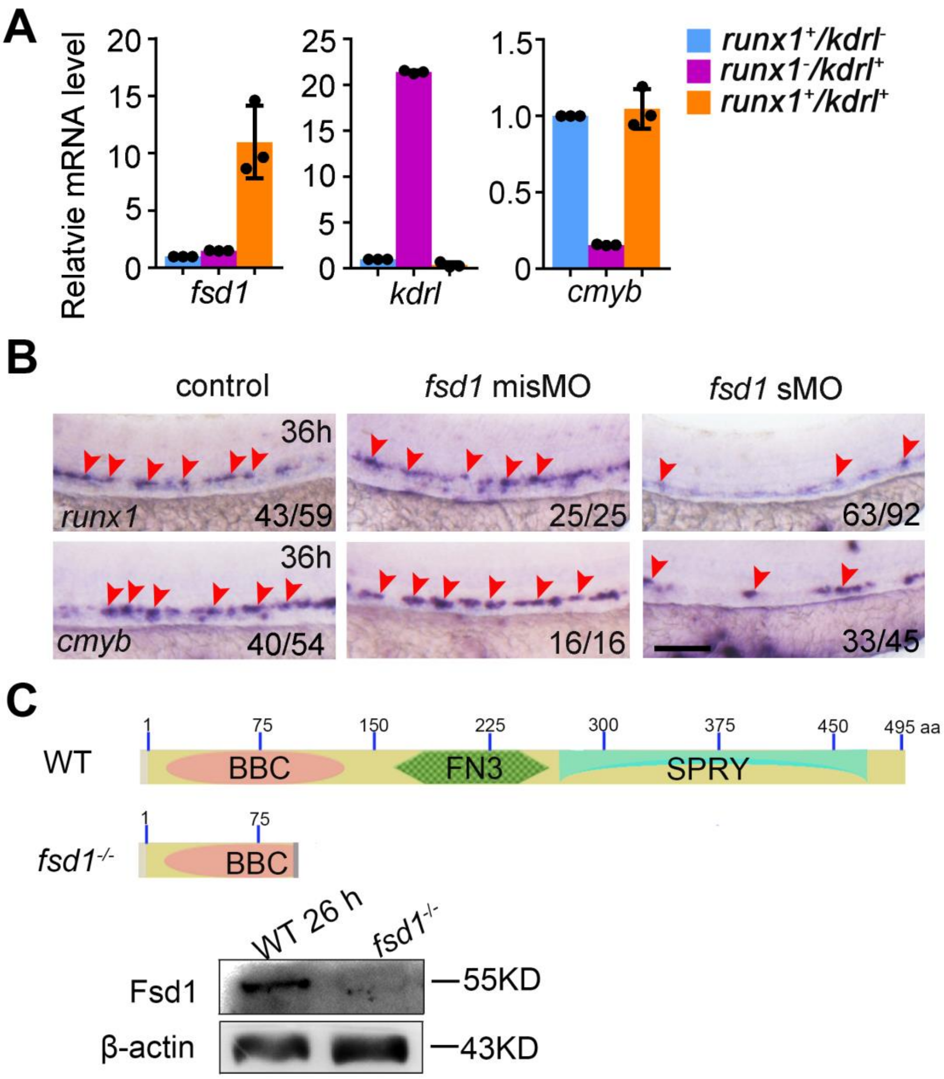

Supplementary Figure 3. HSPC development in *fsd1* sMO-injected embryos and the generation of *fsd1* genetic mutant. (A) Expression levels of *fsd1*, *kdrl* and *cmyb* in *kdrl*$^{+}$*runx1*$^{+}$ cells in the AGM at 26 hpf. The cells (*kdrl*$^{+}$/*runx1*$^{+}$, *kdrl*$^{+}$/*runx1*$^{-}$, *kdrl*$^{-}$/*runx1*$^{+}$) were sorted by fluorescence activated cell sorting from the dissected trunk region of Tg (*kdrl*:mCherry/*runx1*:en-GFP) embryos. n=3 biological replicates. (B) Expression pattern of *runx1* and *cmyb* in the AGM region of *fsd1* sMO-injected and *fsd1* misMO-injected embryos at 36 hpf. Red arrowheads mark expression of HSPC markers *runx1* and *cmyb*. Scale bar, 100 µm (C) Graphic representation of wild type (WT) and truncated Fsd1 protein (upper panel) and protein level of Fsd1 by western blotting (lower panel) in wild type and *fsd1* mutants at 26 hpf.

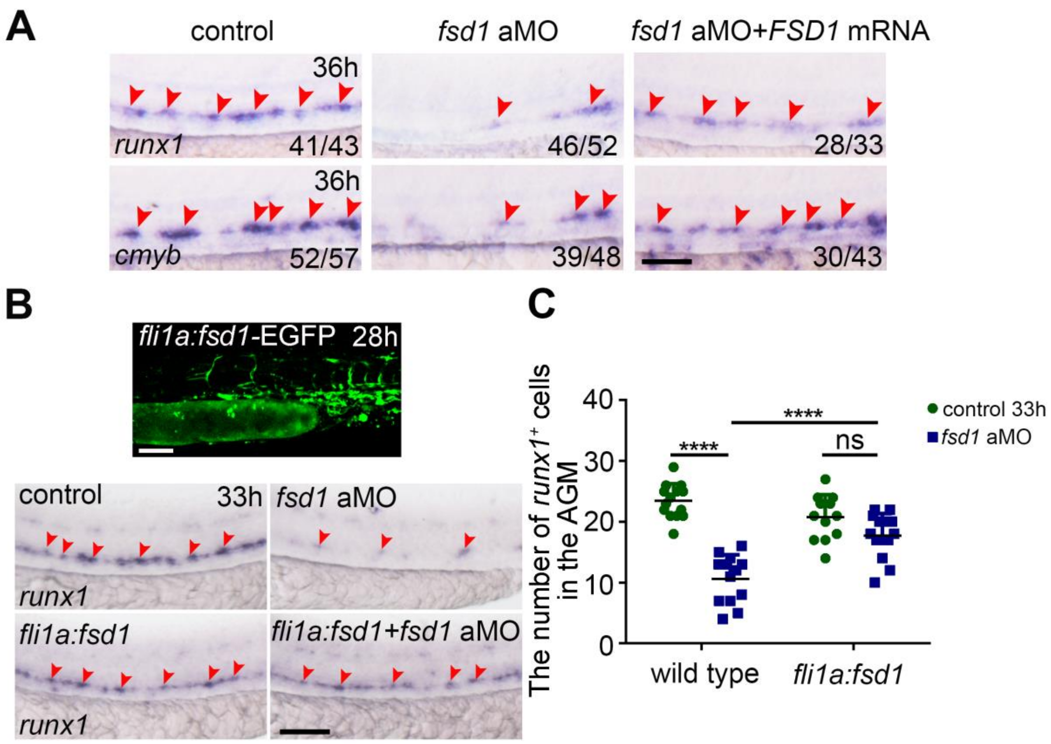

Supplementary Figure 4. *fsd1* overexpression can rescue the defective HSPC development. (A) The expression of *runx1* and *cmyb* (red arrowheads) in control, *fsd1* morphants, and *fsd1* morphants injected with *fsd1* human mRNA. (B) Confocal imaging of embryos injected with *fli1a*:*fsd1*-EGFP plasmid at 28 hpf. WISH experiment presents the expression of HSPC marker *runx1* (red arrowheads) in control, *fsd1* morphants, *fli1a*:*fsd1*-EGFP plasmid injected embryos and *fsd1* morphants injected with *fli1a*:*fsd1*-EGFP plasmid. (C) Quantification of *runx1*$^{+}$ cells in (B). Data represent the analysis results of two-way ANOVA-Tukey test for multiple comparisons. Error bars, mean ± s.d., n=14, 13, 12, 14 embryos, ****$P < 0.0001$. ns, non-significant. Scale bars, 100 μm.

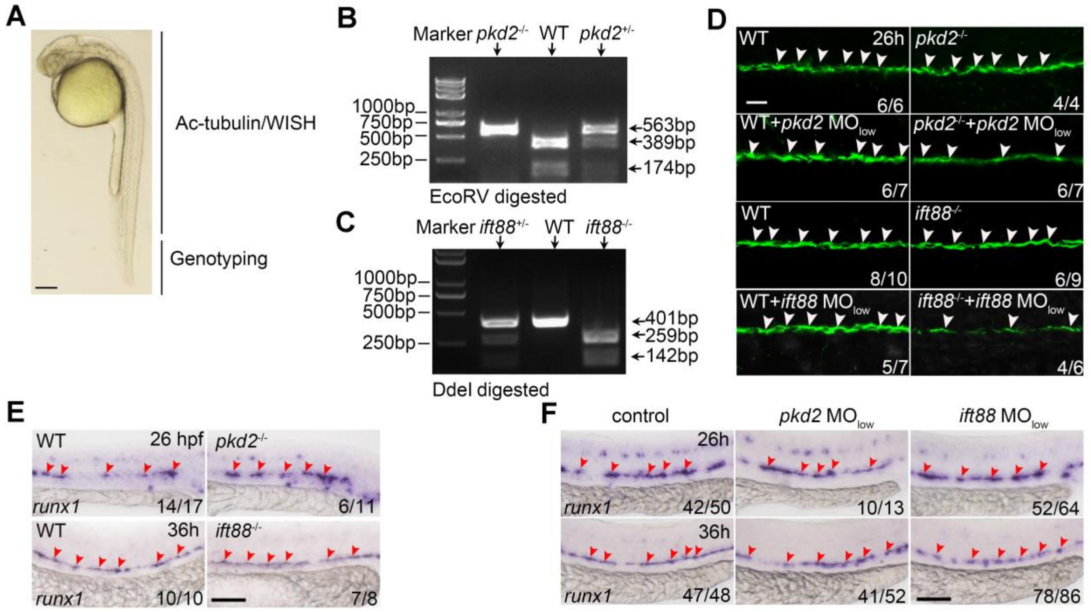

Supplementary Figure 5. Characterization of HSPC development in *pkd2* and *ift88* mutants. (A) A diagram of experiment design. The tail region was used for genotyping. The head and trunk region of embryos were used for Ac-tubulin staining or WISH experiments for examining the cilia formation or HSPC phenotype. (B, C) The genotyping results distinguished *pkd2* or *ift88* mutants (-/-) from their wild type or the carrier. (D) Ac-tubulin staining results showed that the cilia (white arrowheads) in pronephric duct were normal in *pkd2* or *ift88* mutants, compared to wild type siblings. In contrast, when a sub-effective dose of *pkd2* or *ift88* MO was injected into the corresponding mutants, the cilia were affected. (E, F) WISH analysis of the HE and HSPC marker *runx1* (red arrowheads) in *pkd2* or *ift88* mutants (E) and morphants (F). Scale bars, 100 μm in (A), (E), and (F); 10 μm in (B).

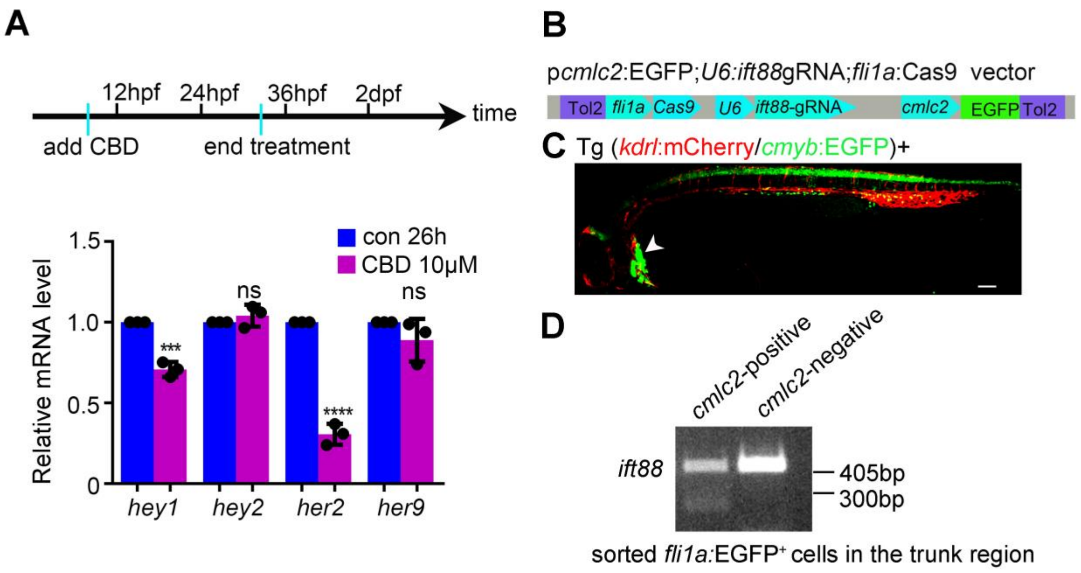

Supplementary Figure 6. CBD treatment and injection with the *fli1a*:*ift88*-cKO plasmid. (A) The illustration of time course for CBD treatment (from 10 hpf to 31 hpf) and qPCR analysis of Notch target genes in control and CBD-treated embryos at 26 hpf. Error bars, mean ± s.d., ***$P < 0.001$, ****$P < 0.0001$, Student's *t*-test. n=3 biological replicates. (B) A brief diagram of *fli1a*:*ift88*-cKO construct. The *cmlc2* promoter-driven EGFP expression was used as a selective marker, and *fli1a* (endothelial cell) promoter-driven Cas9 cassette together with the *U6* promoter-driven *ift88* guide RNA is to achieve endothelial tissue-specific deletion. (C) Cmlc2:EGFP-positive embryos (white arrowheads) indicate that the *fli1a*:*ift88*-cKO vector expressed in Tg(*kdrl*:mCherry/*cmyb*:EGFP) transgenic line. Scale bar, 100 μm. (D) T7E1 assay analysis showing genomic mutation of *ift88* induced by *fli1a*:*ift88*-cKO vector injection. The genomic DNA was extracted from sorted *fli1a*$^+$ cells from dissected trunk region of Tg (*fli1a*:EGFP) embryos with or without the expression of EGFP in the heart at 26 hpf and used for T7E1 assay.

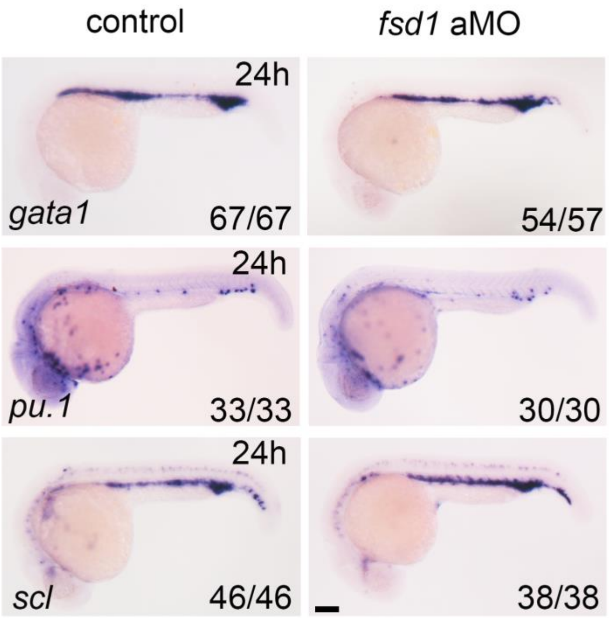

Supplementary Figure 7. Primitive hematopoiesis is normal in *fsd1* morphants. Expression of primitive erythrocyte markers, *gata1* and *scl*, and myeloid cell marker *pu.1* in control and *fsd1* morphants at 24 hpf. Scale bar, 100 µm.

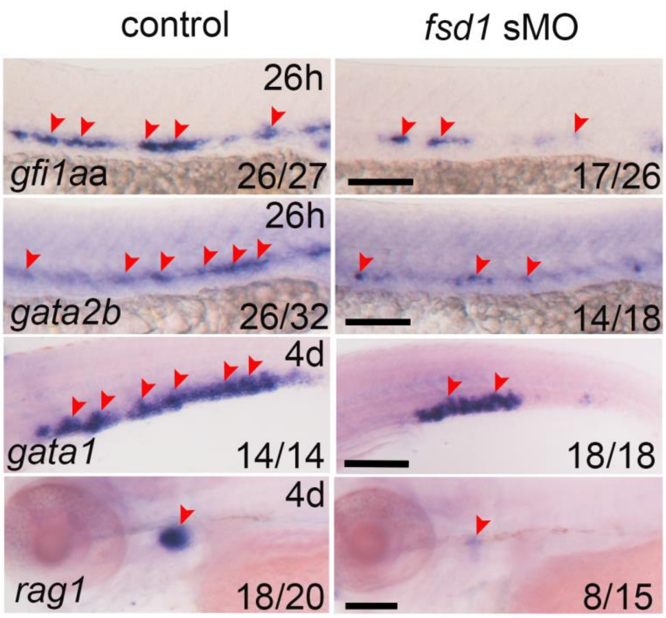

Supplementary Figure 8. The HE specification and HSPC derivatives are affected in *fsd1* sMO-injected embryos. Expression pattern of HE markers (*gfi1aa*, *gata2b*) in the AGM region at 26 hpf, erythroid lineage marker *gata1* in the CHT region and T cell marker *rag1* in the thymus region at 4 dpf. Red arrowheads mark hematopoietic cells. Scale bars, 100 μm.

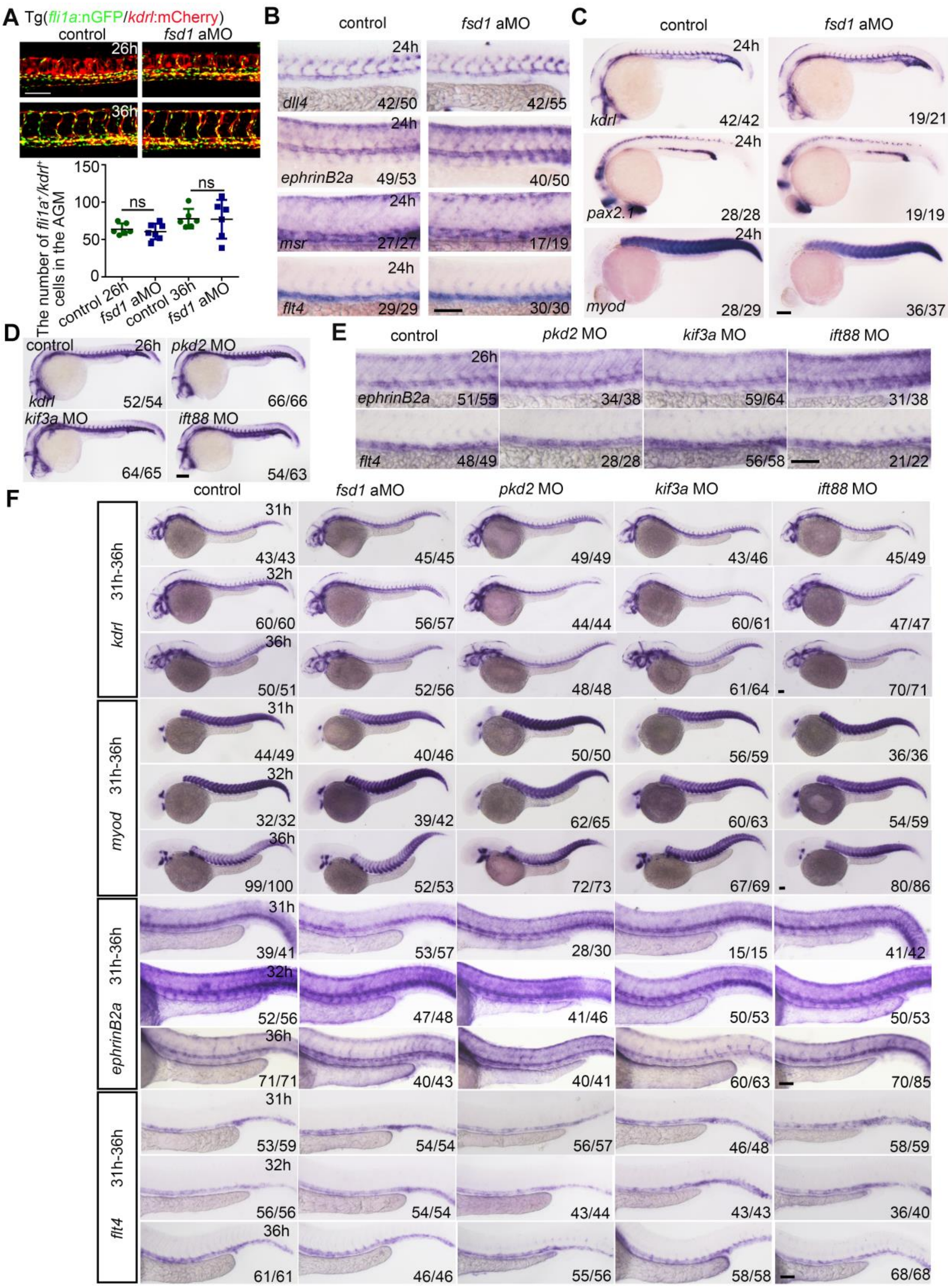

Supplementary Figure 9. The blood vessel and niche cells are normal in cilia-impaired embryos. (A) Imaging of *fli1a:*nGFP^{+}/*kdrl*:mCherry^{+} cells in blood vessels in the AGM region in control and *fsd1* morphants by analyzing Tg(*fli1a*:nGFP/*kdrl*:mCherry) line with quantification (lower panel). Error bars, mean ± s.d., n=6, 7, 6, 6 embryos. ns, non-significant, Student's *t*-test. (B, C) WISH results show the expression pattern of arterial markers *dll4*, *ephrinB2a*, venous markers *msr*, *flt4*, pan-vascular marker *kdrl*, pronephric duct marker *pax2.1* and somitic marker

myod in control and *fsd1* morphants. (D, E) WISH analysis of *kdrl*, *ephrinB2a* and *flt4* in control and cilia-impaired embryos at 26 hpf. (F) WISH analysis of *kdrl*, *myod*, *ephrinB2a* and *flt4* in cilia-impaired embryos at 31-36 hpf. Scale bars, 100 μm.

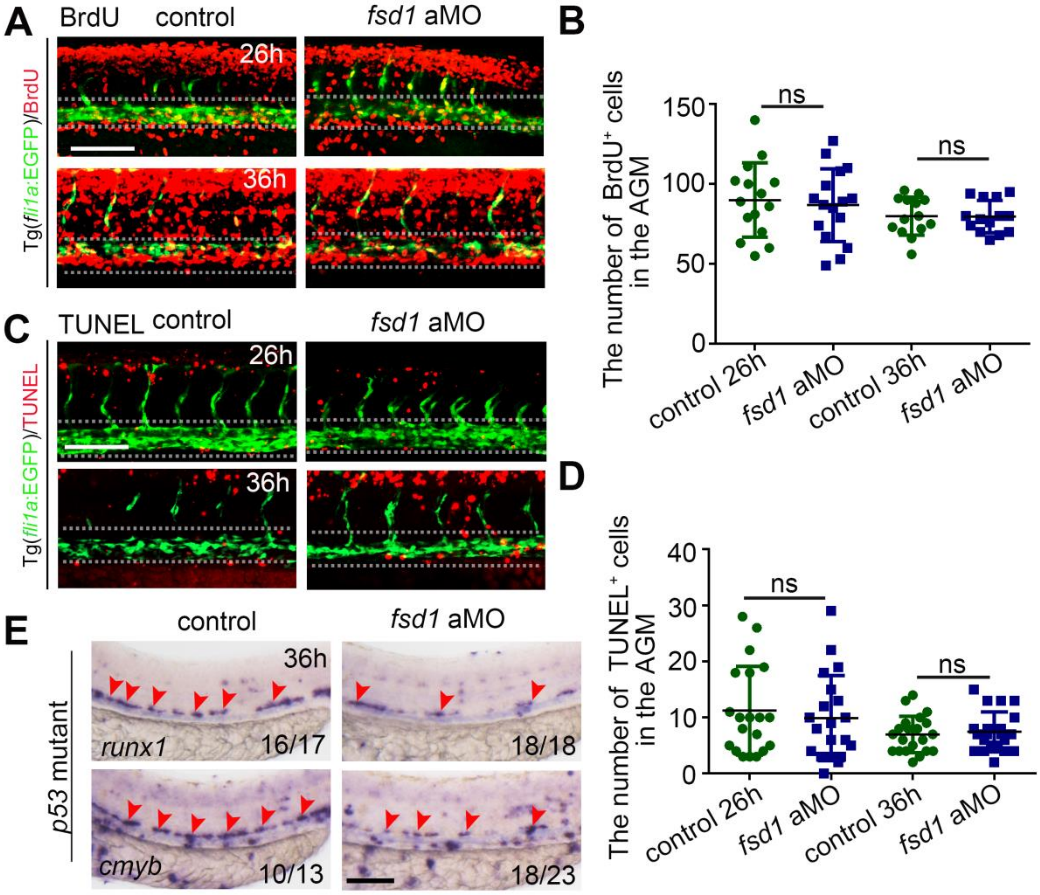

Supplementary Figure 10. The proliferation and apoptosis of ECs are normal in *fsd1*-deficient embryos. (A) BrdU assay in Tg(*fli1a*:EGFP) embryos after knocking down *fsd1* at 26 hpf and 36 hpf. Gray dashed lines indicate the proliferative *fli1a*$^+$ cells. (B) Quantification of proliferative *fli1a*$^+$ cells in control and *fsd1*-deficient embryos. Error bars, mean ± s.d., n=15, 16, 14, 14 embryos. ns, non-significant, Student's *t*-test. (C) TUNEL staining in Tg(*fli1a*:EGFP) embryos after knocking down *fsd1* in blood vessels in the AGM region in control and *fsd1* morphants at 26 hpf and 36 hpf. Gray dashed lines indicate the apoptosis of *fli1a*$^+$ cells. (D) Quantification of apoptotic *fli1a*$^+$ cells in (C). Error bars, mean ± s.d., n=20, 20, 21, 21 embryos. ns, non-significant, Student's *t*-test. (E) Expression of HSPC markers, *runx1* and *cmyb*, in the AGM region in control and *fsd1* morphants in *p53* mutant line. The red arrowheads mark HSPCs in the AGM region. Scale bars, 100 μm.

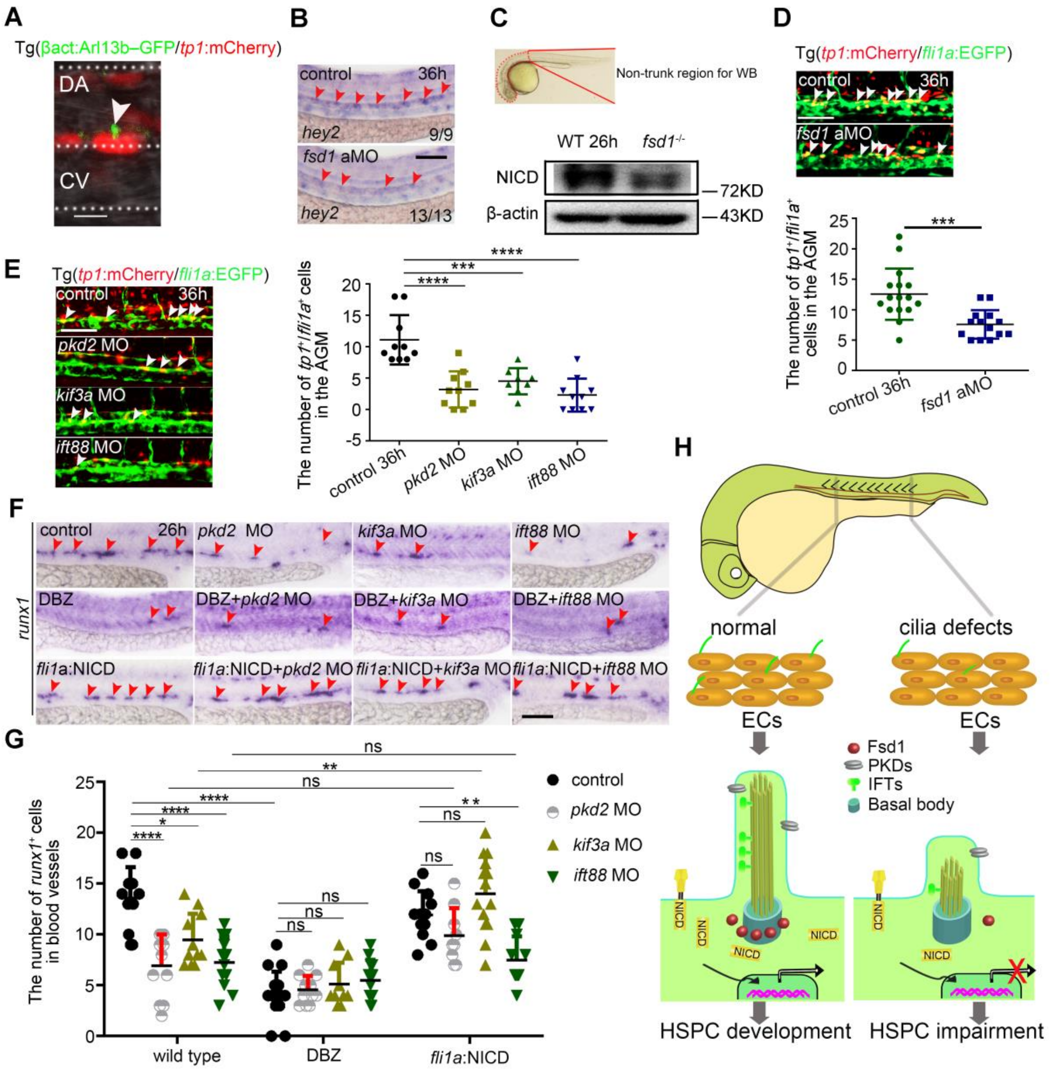

Supplementary Figure 11. Notch signaling acts downstream of primary cilia. (A) Confocal imaging of cilia in Tg(*tp1*:mCherry/βact:Arl13b–GFP) line at 26 hpf. Gray dashed lines mark blood vessels. White arrowhead indicates primary cilia in *tp1*$^{+}$ cells in the ventral wall of DA. (B) WISH result of *hey2* (red arrowheads) in control and *fsd1* morphants at 36 hpf. (C) Protein level of NICD by western blotting in wild type and *fsd1* mutants at 26 hpf. Red dashed line indicates the non-trunk region. (D) The number of *tp1*:mCherry^{+}/*fli1a*:EGFP^{+} cells (white arrowheads) in the AGM region in control and *fsd1* aMO injected embryos (upper panel) with quantification (lower panel) at 36 hpf. Error bars, mean ± s.d., n=14, 16 embryos, ***P < 0.001, Student's *t*-test. (E) Confocal imaging of Tg (*tp1*:mCherry/*fli1a*:EGFP) embryos in the AGM region in control and cilia-impaired embryos at 36 hpf with quantification. White arrowheads mark *tp1*:mCherry^{+}/*fli1a*:EGFP^{+} double positive cells. Data represent the analysis results of one-way ANOVA-Dunnett test. Error bars, mean ± s.d., n=10, 10, 8, 10 embryos, ***P < 0.001, ****P <

0.0001. (F) Expression of *runx1* (red arrowheads) in cilia-impaired embryos with DBZ treatment, or *fli1a*:NICD-EGFP co-injection. Red arrowheads indicate *runx1*$^+$ cells at 26 hpf. (G) The quantification of *runx1* expression in (F). Data represent the analysis results of two-way ANOVA-Tukey test for multiple comparisons. Error bars, mean ± s.d., n=12, 11, 11, 12, 18, 11, 10, 12, 12, 9, 14, 11 embryos. ns, non-significant,**P* < 0.05, ***P* < 0.01, *****P* < 0.0001. (H) Working model of the role of primary cilia in HSPC development through transducing Notch signaling. Scale bars, 10 μm in (A); 100 μm in (B), (D), (E) and (F).

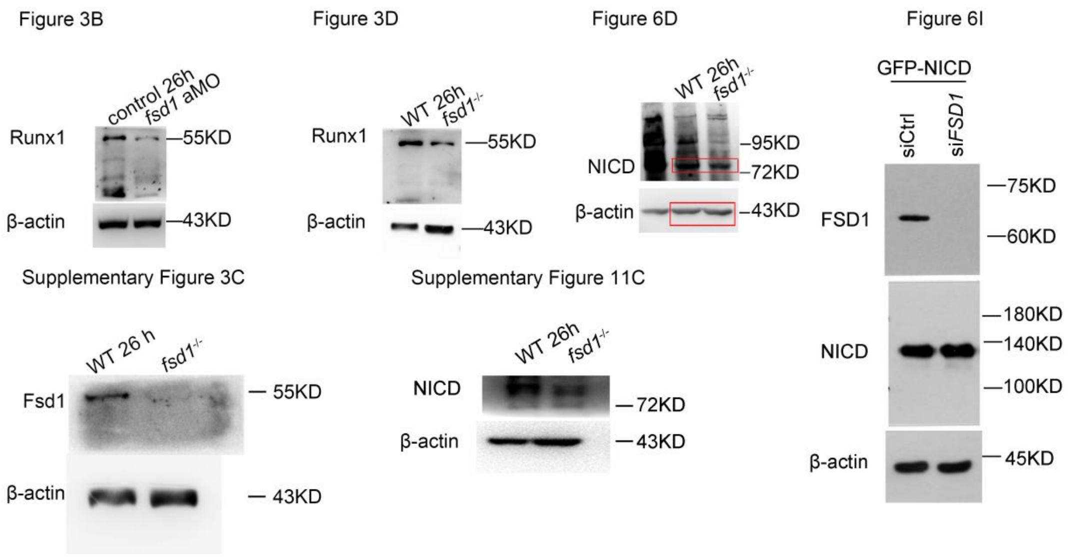

Supplementary Figure 12. Uncropped scans of blots. The uncropped images of blots shown in Figure 3, 6, Supplementary Figure 3 and Supplementary Figure 11 are listed.

Supplementary Tables

Supplementary Table 1. Primers for mutant genotyping.

Genes	Sequence (from 5’ to 3’)
fsd1 mutant	F: CTCAATACCTGAATTAGTGGGA<br>R: TTGTTAGAGCTGCAATAGCA
ift88 mutant	F: GCAAATTAGTGCATATAACGCCTC<br>R: CCA ATGTTCTGCATGATCTTTATCC
pkd2 mutant	F: TCTTTTGCTCACAGTTTCACG<br>R: AAAAACAGCCCCAACATAACG

Supplementary Table 2. MOs used in this study.

MOs	Sequence
fsd1 aMO	5’-ACTCCTTCTGGTCGTCCATGCTGTC-3’
fsd1 sMO	5’-TGGCCTCCTGTAGTGTTTACCTCTA-3’
fsd1 misMO	5’-ACTACTTATGGTAGTCCATACTATC-3’
kif3a MO	5’-GTCCAGCTTATTGCTCGGCATTATC-3’[1]
pkd2 MO	5’- AGGACGAACGCGACTGGAGCTCATC-3’[2]
ift88 MO	5’- CTGGGACAAGATGCACATTCTCCAT-3’[3]

Supplementary Table 3. Primer sequence for *fli1-ift88*-cKO plasmid construction and genotyping[8].

Name	Sequence	
fli1-ift88-cKO-pcr-F	5’- GTAATGGCTGCCAGTTTAC -3’	genotyping
fli1-ift88-cKO-pcr-R	5’- TACGTCAAACCTGCATGATG-3’	
fli1-ift88-cKO-gRNA-F	5’-GGCTGACCGCTATGCAGAGCgt-3’	plasmid construction
fli1-ift88-cKO-gRNA-R	5’-GCTCTGCATAGCGGTCAGCCga-3’	

Supplementary Table 4. qPCR primers used in this study.

Gene name	Sequence (from 5’ to 3’)	References
fsd1	F: TGACACGCGCTACATGA<br>R: CCTGAACAGCAACCTTCC	This study
kdrl	F:GAGAACGGAACCAACAAGATCCACGAG<br>R:CCCTCCAGCAGAACTGACTCCTTAC	
her1	F:TGGAATTGGCTGTTGAGTAT<br>R:CCTTGTAGATTGGATTAGATGG	
actin	F: GCTGTTTTCCCCTCCATTGTT<br>R: TCCCATGCCAACCATCACT	[4]
hey2	F: ATTGATGTGGGCAGCGAGAA<br>R: TGGGATGTGGTGGATGTGGA	
notch1a	F: CGGGCCTGACGGATTCAC<br>R: GGACTCCAGCAGACGTTTAGC	

her2	F: CAATGGCACCAACTGTCT<br>R: CTTGAATATCCGTCAGCATAG	5
her5	F: GGAGAGTGATGAGGATGTG<br>R: GATGCTGCTGTGATGGAT	
her9	F: CAGCCACGGACGGACAGTTT<br>R: AACGCCCGAGAAGGAGGTCA	
hey1	F: TTTGATGCTCACGCTCTGGC<br>R: ACCTGCTGAGATGGGACAAG	6
cmyb	F: TGATGCTTCCCAACACAGAG<br>R: TTCAGAGGGAATCGTCTGCT	7

Supplementary Table 5. siRNA used in this study.

Name	Sequence
siCtrl	5’-UUCUCCGAACGUGUCACGUAA-3’
si*FSD1*	5’-GCAGGAUAUCAAGGCUCGCGAGAAA-3’

Supplementary References

1. Pooranachandran, N. & Malicki, J.J. Unexpected Roles for Ciliary Kinesins and Intraflagellar Transport Proteins. *Genetics* **203**, 771-785 (2016).
2. Sun, Z. *et al.* A genetic screen in zebrafish identifies cilia genes as a principal cause of cystic kidney. *Development* **131**, 4085-4093 (2004).
3. Kramer-Zucker, A.G. *et al.* Cilia-driven fluid flow in the zebrafish pronephros, brain and Kupffer's vesicle is required for normal organogenesis. *Development* **132**, 1907-1921 (2005).
4. Zhang, C. *et al.* m6A modulates haematopoietic stem and progenitor cell specification. *Nature* **549**, 273-276 (2017).
5. Wei, Y.L. *et al.* Ncor2 is required for hematopoietic stem cell emergence by inhibiting Fos signaling in zebrafish. *Blood* **124**, 1578-1585 (2014).
6. He, Q. *et al.* Inflammatory signaling regulates hematopoietic stem and progenitor cell emergence in vertebrates. *Blood* **125**, 1098-1106 (2015).
7. Xue, Y. *et al.* The Vascular Niche Regulates Hematopoietic Stem and Progenitor Cell Lodgment and Expansion via klf6a-ccl25b. *Developmental cell* (2017).
8. Chen, X., Gays, D., Milia, C. & Santoro, M.M. Cilia control vascular mural cell recruitment in vertebrates. *Cell reports* **18**, 1033-1047 (2017).
